# Health behaviours of young mothers: Implications for health promotion and cancer prevention

**DOI:** 10.1177/0017896917745106

**Published:** 2017-12-20

**Authors:** Lucy Hackshaw-McGeagh, Kimberly Jamie, Rhona Beynon, Roisin O’Neill

**Affiliations:** a NIHR Bristol Biomedical Research Centre Nutrition Theme, University of Bristol, Bristol, UK; b School of Applied Social Sciences, Durham University, Durham, UK; c Population Health Sciences, Bristol Medical School, University of Bristol, Bristol, UK; d Centre of Excellence for Public Health Northern Ireland, Queen’s University Belfast, Belfast, UK

**Keywords:** Cancer prevention, focus group, health behaviours, photo-elicitation, young mothers

## Abstract

**Objective:**

Evidence suggests that younger mothers engage in poorer health behaviours, resulting in increased cancer risk. We aimed to better understand the health behaviours of younger mothers and the factors that influence their lifestyle choices, in order to improve cancer prevention within this population.

**Methods:**

A multiple focus group, photo-elicitation-aided approach was used, in which young mothers (*n* = 27; aged 16–24 years) were provided with cameras and asked to capture ‘a week in your life’. Photographs were developed and participants invited to an initial focus group where photographs were used to elicit discussion, exploring participants’ health behaviours. Data were thematically analysed particularly identifying themes relating to barriers and facilitators of positive health behaviours. Participants were later invited to participate in a second focus group, to explore and validate identified themes further.

**Results:**

Themes emerged from the data relating to (1) the mothers’ personal perceptions of health, (2) health-related behaviours and (3) beliefs about cancer and its causes. Barriers to positive health behaviours included a lack of money, childcare and cookery skills; facilitators included the social media, commercial weight loss programmes and local community organisations.

**Conclusion:**

Study findings provide insight into the health behaviours and life choices of young mothers. They help illustrate health perceptions in relation to cancer risk, providing an understanding of how their daily routine and circumstance influence young women’s decisions and lifestyle behaviour choices and highlighting barriers to, and facilitators of, positive health behaviours. Data hold potential to inform future health-related research among young mothers, particularly relating to cancer prevention intervention.

## Introduction

The significance of lifestyle factors in the development of cancer is well established (Parkin et al., 2011; World Cancer Fund, 2007). Estimates suggest that 42% of cancer cases in the United Kingdom each year are linked to a combination of 14 major lifestyle factors (Parkin et al., 2011). Socioeconomic differences have been identified in many of these contributory risk factors (Mackenbach et al., 2008; Wardle et al., 2003). For instance, those living in areas with a lower socioeconomic profile are more likely to have tried smoking, to be physically inactive and to eat a high fat diet (Bambra, 2016; Hanson and Chen, 2007; Wardle et al., 2003). Awareness of cancer symptoms is also lower among socially and economically deprived populations (Redeker et al., 2009), and people living in disadvantaged communities are less likely to utilise cancer-screening services (Ward et al., 2004). Therefore, health outcomes are not just the result of individual decision-making but are rooted in structural inequalities, whereby deprived communities have poorer access to health services, poorer health education and increased housing and financial pressures, which, in turn, may lead to poor health behaviours.

Young mothers represent one group who may be at particular risk of engaging in health behaviours that may lead to cancer and poorer health outcomes. The reasons for this are threefold: first, there is a strong association between teenage pregnancy and social deprivation (Imamura et al., 2007), meaning that younger mothers may struggle financially, live in poorer housing and have lower educational attainment (Hobcraft and Kiernan, 2001; Letourneau et al., 2004); second, cancer risk behaviours like smoking and physical inactivity often begin in youth (Wardle et al., 2003); and third, young mothers may leave school early or move away from the parental home, leaving them at risk of isolation due to inadequate social support (Evans and Slowley, 2010; Letourneau et al., 2004).

Despite being a high-risk group, there is a notable absence of young mothers from public health research, especially cancer research. While research focusing specifically on young mothers tends to foreground the health outcomes of their children, research on young people more generally often subsumes young mothers within a homogeneous body of ‘young people’, not recognising the complexities and specificities of young motherhood. Moreover, young mothers may be understood as a ‘hard-to-reach’ group (Sydor, 2013), given their assumed relative socioeconomic disadvantage, disengagement from services and authorities and perceived lack of trust of researchers. Although we take issue with this simplistic characterisation of young mothers (and indeed other populations) as ‘harder to reach’, structural inequalities can make research with relatively disadvantaged groups challenging to undertake.

Addressing preventable risk factors in young mothers is critical to reduce cancer risk and improve health outcomes. In this study, we aimed to better understand the health behaviours of younger mothers and the factors that influence their lifestyle choices to both inform cancer prevention in this population and highlight the intersections between wider structures of inequality and social determinants of health and their everyday health-related behaviours.

## Methods

### Population and recruitment

In this study, we defined young mothers as those who had at least one child at age 21. Women were recruited from three areas of the United Kingdom – in Belfast, Northern Ireland; in Bristol in South-West England; and in the Middlesbrough area in North-East England. Each of these localities has varying levels of deprivation. For example, Belfast hosts 51 areas which are listed in the most 10% deprived areas in Northern Ireland (NISRA, 2010) and has high unemployment rates at 5.4% (UK average 4.6%; NISRA, 2017); Bristol hosts 42 areas which are listed in the most 10% deprived areas nationally (Bristol City Council, 2015), with one quarter of all children living in poverty; and Middlesbrough has one of the highest teenage pregnancy rates in England and Wales at 33.8 in every 1,000 young women (ONS, 2015).

**Figure 1.**
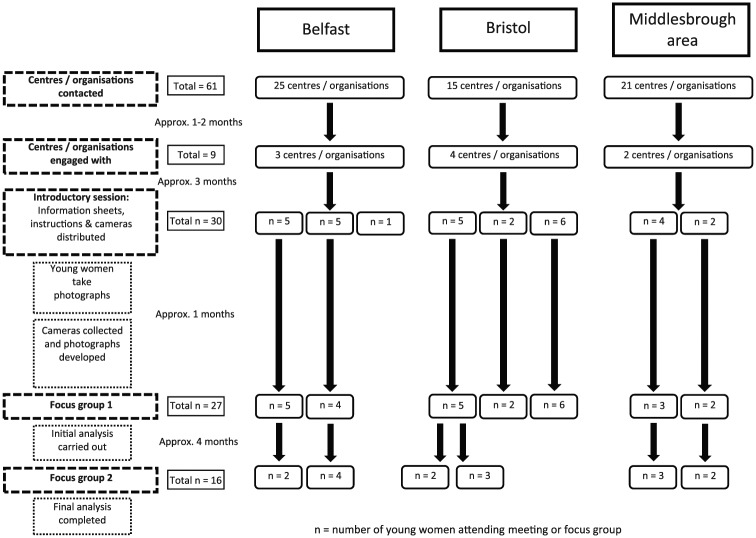
Breakdown of organisations and participants involved in the research by area.

To access young mothers, we approached local Children’s Centres and other organisations to act as gatekeepers. This proved to be challenging due to understaffing, incorrect publicly available contact details and lack of interest in some instances. Of the 61 organisations we initially approached, we received responses from 9 of them (Figure 1) and arranged meetings with 8, all of which agreed to take part. Direct invitation flyers providing an overview of the project were then sent from the organisations to potential participants on behalf of the research team. These flyers included details of the project, an explanation of what participants would be asked to do, an invitation to an information session and a photograph of the local researcher. To increase participation and retention, the organisation’s logo was also clearly visible.

Potential participants were then invited to an introductory session to find out more about the project (*n* = 30). Information sheets were provided, questions answered and fully informed consent given. The content and language of the information sheets were developed with input from support workers at the organisations to ensure they were appropriate to the young women. Three of the women who attended this introductory session decided not to participate further. Reasons for this were not provided. The breakdown of recruitment numbers and participant pathway through the research can be seen in Figure 1.

### Photo-elicitation methodology

Participants were provided with a disposable camera and asked to take photographs during the following week which they felt demonstrated their everyday life as a young mother. We did not ask that these photographs pertained to health but to everyday life and typical activities more broadly. We felt that directing participants to generate health-specific pictures would not have provided a full illustration of their everyday lives and the contexts in which health-related decisions are made. By giving participants autonomy over the content of the pictures, we aimed to generate a more holistic perspective. The photographs were intended as stimulus material for focus group discussions and were not analysed separately. Photographs were used to (1) give participants a degree of ownership of the project, (2) engage the participants deeply in the project and (3) informally begin conversations and as a prompt for discussion. For example, asking participants to describe what was happening in a photograph in which they were playing with their children elicited a more natural discussion than directly asking participants about their interaction with their children without visual prompts. Moreover, we were concerned that direct questioning without participant-generated prompts might be perceived as confrontational, conflating the focus group questioning with the approach often used by social services departments, which many of the women were wary of. Similar methodologies have been used in other contexts, such as within the lesbian, gay, bisexual and transgender (LGBT) community (Johnson, 2007) and in homeless populations (Radley et al., 2005). In each of these instances, photo-elicitation research was used as a tool to reduce pre-established barriers or to access harder to reach minorities, enabling participants to reflect on their everyday experiences and enabling researchers to access information that might otherwise be out of reach (Reavey, 2012). Photo-elicitation research has been shown to grasp detailed accounts of experience (Cappello, 2005) and to capture private experiences that might otherwise be forgotten about, in an unobtrusive manner (Young and Barrett, 2001).

### Focus groups and data analysis

Participants were invited to participate in Focus Group 1. Here, the photographs were displayed to the group, with the agreement of the photographer; if a young woman chose not to share an image, it was put aside and not viewed by the other members of the group. Participants were asked to discuss the content and what it illustrates regarding their everyday life as a young mother. These were informal discussions started by discussing specific photographs and guided by a topic guide where necessary (see Supplementary Material, Appendix 1). One example of how the focus group conversation began is shown below:

Interviewer: So do you want to just take it in turns to go through and talk about your photos; I’ve looked at the photos and I have a few questions I want to ask you about them as you go. So who wants to go first?

Neave: Em, this is my everyday lifestyle, looking after my kids, going to work and living off diet coke.

Interviewer: When you say you’re living off it, how many tins a day would you drink?

Neave: 12 tins a day easily. On a good day. (Focus Group 1 – Belfast)

In most cases, groups were constructed around pre-existing networks, but in some cases participants were unknown to each other. Focus groups consisted of two to six participants and took place within the local organisations. They lasted between 90 and 150 minutes and were audio recorded and transcribed verbatim.^1^ While a topic guide was used to direct conversation at appropriate moments, the discussions were flexible, primarily focusing on topics of interest and importance to the participants. Focus groups are a long-standing, widely used methodology and have been repeatedly shown to successfully facilitate interaction between participants resulting in rich, naturally occurring, data, particularly where participants are known to each other resulting in ‘collective remembering’ (Krueger and Casey, 2015).

Transcripts were then analysed thematically using the framework approach (Ritchie et al., 2014). This method of analysis consists of five stages: familiarisation, identification of thematic coding framework, indexing, charting, mapping and interpretation. For the purpose of this article, all transcripts from each focus group were analysed by L.H.-M., with 20% of transcripts being analysed by both L.H.-M. and R.B. to ensure validation triangulation of themes. Lead researchers from each research site (R.O.N., L.H.-M. and K.J.) held regular Skype calls to discuss the ongoing analysis. All participants were allocated a pseudonym to ensure confidentiality and, where others were mentioned, all names were changed. Data were analysed using NVivo software.

Following the analysis of Focus Group 1, participants were invited to Focus Group 2 to explore themes further and gain feedback on our initial analysis and interpretation of results, improving the dependability and credibility of the analysis. A total of 16 of the 27 original participants participated in Focus Group 2. In the current analysis, only two quotes from Focus Group 2 were used; the remainder of quotes were all taken from Focus Group 1, given that they provided the majority of rich exploratory data.

Participants were reimbursed for their travel; lunch and childcare were provided where necessary. Bristol participants also received a £20 gift voucher, funded by a local grant (NIHR Biomedical Research Unit). Data were collected with informed consent, and the University of Durham Ethics Committee approved the study. The University of Bristol’s Secretary’s Office reviewed the research proposal, and participants were required to sign a document to certify that ownership of all the photographs taken rested with the University of Bristol. Participants had the right to remove any photographs from group discussion and were offered as many copies of each photograph as they wished to keep.

## Results

In total, 27 mothers, from three UK areas, Belfast (*n* = 9), Bristol (*n* = 13) and the Middlesbrough area (*n* = 5), participated in this research. Participants ranged in age from 16 to 24 years at the time of focus group, had between one and three children, who were aged between 2 weeks and 6 years and were all of White British or Irish background. Although we did not collect specific demographic details, most participants were from neighbourhoods with a low socioeconomic profile and had experienced, or were currently experiencing, multiple deprivation and challenges, such as single parenthood, precarious housing, unemployment, debt and domestic violence.

The content of the photographs differed between participants but captured daily activities such as trips to the park with their children, events involving family and friends, foodstuffs, alcoholic drinks and cigarette packets. As mentioned, the photographs themselves were not used as data or analysed, but instead employed as a tool for eliciting discussion. To add context to the research, however, we have included a selection of photographs in the Supplementary Appendix. These photographs have been reproduced with the permission of the young mother who took each photograph (see Supplementary Material, Appendix 2). The images initiated in-depth participant-led conversations about their daily lives and, when necessary, researchers guided discussion towards aspects relating to health behaviours and cancer beliefs.

### Definition and personal perception of health

Participants were asked what health meant to them. For some, a range of typically health-related behaviours were described, for example, low alcohol and tobacco consumption, healthy diet and physical activity behaviours, suggesting an awareness of factors which contribute to good health. When asked whether they believed themselves to be healthy, there was an almost unanimous negative response; many laughed at the concept of seeing themselves as healthy:

Interviewer: If I asked you would you describe yourself as healthy?

Izzi: No

Emma: No

Gemma: No [laughter]. (Focus Group 1 – Bristol)

Some participants provided explanations for why they felt their behaviour was unhealthy, whereas others suggested they understood what it meant to be healthy, but this was not consistent or feasible with their lifestyles:

Interviewer: Would you say you are healthy?

Chloe: [laughter]

Daisy: No. I eat junk food, put loads of weight on, smoke loads and don’t exercise that much. (Focus Group 1 – Bristol)

Interviewer: How would you describe health?

Jade: When I think of healthy I think of eating fruit and doing exercise whereas that’s just not the reality in my life. (Focus Group 1 – Middlesbrough area)

While participants were well versed in what constitutes positive health behaviours, the integration of these behaviours into their everyday lives was felt to be unfeasible both because of everyday pressures such as a lack of time to prepare meals and broader social structures such as the expense of healthy food compared to unhealthy alternatives.

### Health-related behaviours

There was much discussion around the typical health behaviours of the participants, particularly in relation to diet, physical activity, smoking and alcohol consumption. Barriers to, and facilitators of, positive health behaviours became apparent within these discussions.

#### Diet

It was generally suggested that participants did not consume a healthy diet. The two key barriers preventing consumption of a healthy diet were (1) lack of time to cook meals and (2) the cost of healthy foods/ingredients to cook a meal from scratch, both of which are compounded by the availability of cheap convenience foods and takeaway outlets:

Neave: It actually kills me to make a healthy dinner because you see by the time you buy all your stuff for a healthy dinner, you could have had two takeaways, that’s what way I think of it!

Interviewer: So cost is a big thing?

Robyn: They are all saying ‘eat healthy’ but it’s too dear! (Focus Group 2 – Belfast)

Some participants described consuming takeaway foods almost every day:

Neave: Tell the truth. How many takeaways would you have on an average week?

Olivia: 4 or 5

Neave: 5 or 6

Olivia: I wouldn’t have a takeaway every day

Neave: But for your lunch you would have a take away wouldn’t you?

Olivia: Yes

Interviewer: So you would have a takeaway every day depending on whether it’s lunch or dinner?

Olivia: Yes

Paisley: She doesn’t do cooking that’s what she is saying. (Focus Group 1 – Belfast)

These data show that everyday decisions about food were made within particular social and economic contexts in which unhealthy takeaway food is more readily available and cheaper than healthy alternatives. While neoliberal discourses of personal responsibility foreground the importance of making healthy choices, participants demonstrated that multiple and intersecting deprivations mediate these choices. Limited cookery skills were noted, particularly in the younger mothers (aged 16–19 years) who often lived at home compared with ‘older’ mothers (early 20s) some of whom discussed trying to cook from scratch:

Kaylee (aged > 19): our new landlord got us a brand new cooker so we’re all like ‘get the Spaghetti Bolognese and everything on’. (Focus Group 1 – Belfast)

Madison (aged 16–19): I don’t know how to cook. (Focus Group 1 – Belfast)

In some cases, membership of weight loss groups such as Slimming World was reported to facilitate the consumption of a healthier diet and encouraged participants to cook healthier meals and avoid takeaway foods:

Neave: If I’m on Slimming World I would only have one takeaway a week. If I’m doing Slimming World I cook from fresh and do things the healthy way but if I’m not on Slimming World I would do chips. (Focus Group 1 – Belfast)

These data suggest that some kind of peer support system for healthy eating may well be of value for young mothers, although interventions such as these are likely to be set against a backdrop of inequality and deprivation.

#### Physical activity

Attendance at gym and exercise classes were generally not feasible due to membership costs, lack of time, as well as additional childcare costs:

Jade: It’s (gym) £28 a month or something like that but you have to pay for the crèche separately … I can’t because it’s so expensive. (Focus Group 1 – Middlesbrough area)

For some participants, walking to the Children’s Centre was the extent of their physical activity. For others, the only exercise that they did involved playing with the children and completion of household chores:

Izzi: I suppose that’s exercise isn’t it? Cleaning the windows [Laughter], Vacuuming. ([Focus Group 1 – Bristol)

Amy: We’re running up and down the stairs every day just to make them go to the toilet is like enough walking for me I think [laughter]. (Focus Group 1 – Bristol)

These accounts of lack of time and money for exercise highlight the need for affordable programmes in areas of high deprivation. Paradoxically, those areas which would benefit most from investment in public exercise spaces, such as swimming pools and gyms, tended to be those in which funding has been most drastically reduced under current UK government austerity measures (Berry and White, 2014).

#### Maintenance of a healthy body weight

Some participants said they did not care about carrying extra weight and, thus, had no desire to modify their lifestyle in order to lose weight:

Brooklyn: I’m overweight and I do not care [Laughter]. (Focus Group 1 – Bristol)

Daisy: It’s like what’s the point? I might as well just get fat and whatever. (Focus Group 1 – Bristol)

Stacey: I don’t care. (If people) don’t like, don’t look, simple. (Focus Group 1 – Belfast)

When body image and the avoidance of putting on weight, or the intention to lose weight were discussed, their main motivation was for aesthetics rather than a desire to be healthy or improve their wellbeing:

Interviewer: What is your motivation for wanting to lose weight?

Neave: Not to be fat … the tyres.

Interviewer: Would you say it’s image or would you say it’s health related?

Neave: Image. Have to look good [laughter] – I’m saying that like I love myself! Do you ever get when you are going somewhere and if you have your spare tyres -as I call them – hanging out? It’s not attractive. (Focus Group 2 – Belfast)

This suggests that while obesity presents a significant health risk, young women are primarily concerned with the impact of weight gain on their body image, reflecting the pressure that young women are under to conform to particular aesthetic models. While this pressure on women is problematic more generally, it is compounded when women are experiencing multiple disadvantages such as poverty and single parenthood. Such intersecting inequalities (Crenshaw, 1989) mean that women may experience multiple stigmas (being overweight, being poor and being a single parent) while lacking access to support or resources to address these issues.

#### Smoking

Almost half (*n* = 13) of all participants smoked, while many of the remaining participants were fervently anti-smoking:

Kaylee: I can’t even stand round a person who is smoking. Most of my family smoke but the minute someone lights up one, I’m halfway up the road.

Participants who did smoke tended to use cigarettes as pain or stress relief. They were often defensive about this behaviour, suggesting they were aware of its detrimental impact on health but justified it as a coping mechanism:

Gemma: If I just have a standard day I smoke like three, and I’d be fine, but when she’s pissy I smoke 10. (Focus Group 1 – Bristol)

Paisley: Kids and stress makes you smoke more. (Focus Group 1 – Belfast)

Leah: It’s a comfort too. And I might have gall stones but sometimes if I have a smoke or if I have a sore head or a pain, it’s like taking a Paracetamol sometimes. (Focus Group 1 – Belfast)

When asked about attempting to quit, many participants said they had tried and failed, with the key barrier to successfully quitting being stress and the temptation of others smoking around them:

Interviewer: What made you go back on them?

Paisley: Everyone smoking around me after my first child. My brother lived in my Mummy’s … he smokes and he used to blow the smoke in my face so I just went back on. (Focus Group 1 – Belfast)

Evidence repeatedly demonstrates that rates of smoking are considerably higher in areas of high economic and social deprivation than in more affluent neighbourhoods (CRUK, 2017). Therefore, smoking ought to be understood not only as a health-related issue but also within a cultural context.

#### Alcohol

In terms of alcohol consumption, some participants suggested they drank less than other women in their age without children, an unsurprising observation due to increased responsibility and, in some cases, a lack of childcare and money:

Kaylee: If I go out to a bar and I see a drink and I think God I could buy a packet of baby wipes or a packet of nappies with that so I hate going out and spending money on drink when you could buy him stuff with it … (Focus Group 1 – Belfast)

In some cases, not drinking due to being a mother seemed to be a cause of upset and had led to isolation and a feeling of being different from peers:

Daisy: I really struggle, like, because we are young we are expected to go out and party and get drunk. Sometimes I’m like ‘I just want to be normal, I just want to go out and get drunk’. (Focus Group 1 – Bristol)

However, other participants stated that when childcare is available and they drank alcohol, this generally involved a form of binge drinking:

Interviewer: Okay. So, you drink – would you say it’s binge drinking at weekends?

Amy: Yeah.

Brooklyn: Every weekend I think is binge drinking. [Laughter]. (Focus Group 1 – Bristol)

This demonstrates that peer pressure and local culture to drink excessively were felt by some young people with children. In instances where they have childcare and time to themselves, young mothers may drink excessively in an effort to ‘get drunk’ and ‘be normal’.

#### The Internet, social media and health

The social media played a significant role in the social lives of participants who often used it to communicate with their friends. However, it also seems to act as a platform for health information and support for both their own and their children’s health:

Neave: A couple of weeks ago George came up in a rash and I put a picture up (on Facebook) to see if anyone else’s child had something like that. (Focus Group 1 – Belfast)

Amy: If one of them is ill they’ll put a picture on Facebook and tag us like ‘what do you think is wrong with her?’ (Focus Group 1 – Bristol)

Asking friends and peers for support via social media platforms such as Facebook is an important strategy in dealing with their own and their children’s health. In many instances, this was the first port of call for advice and a preferred method before visiting a health professional such as a general practitioner (GP) or pharmacist.

Other participants talked about using the Internet for health queries; however, reputable sources of health information were not always used:

Izzi: I use the internet a hell of a lot … temperatures, respiratory rates, anything like that, I type it all in to Google. (Focus Group 1 – Bristol)

The advice provided by friends on Facebook was generally based on their individual past experiences. Young mothers also relied heavily on Google and other general Internet sites such as peer discussion in online chat rooms for health-related advice. In many cases, the information received is not regulated or based on firm scientific evidence.

### Beliefs about cancer and its causes

There was substantial discussion, initiated by the researchers, about participants’ understanding and beliefs about the causes of cancer. Conversations also explored their views concerning cancer prevention messages.

#### Cancer is beyond our control

Many mothers believed that cancer is attributable to bad luck, with little that can be done to prevent it in terms of lifestyle or behaviour change:

Brooklyn: If you are going to get it, you’re going to get it. (Focus Group 1 – Bristol)

Daisy: I just think that must just be unlucky if you get it … Every sort of person has got cancer before, like famous people, non-famous people, athletes, non-healthy people … (Focus Group 1 – Bristol)

A number of participants suggested that everyone has cancer within them, and that it gets ‘triggered’ in some people and not in others.

#### Cancer can be caused by our behaviour

Despite the majority of participants suggesting that cancer is largely beyond individual control, there was some discussion about different behaviours that could lead to or in some cases ‘cause’ cancer:

Daisy: If you smoke too much or you drink too much like bad stuff. Or, if you like, eat too much shitty food and don’t exercise at all, like you literally just sit on your arse all day every day, I think that is when you are most likely to get cancer. (Focus Group 1 – Bristol)

The knowledge demonstrated about the links between cancer risk and lifestyle behaviours was generally poor. In addition, whenever these risk factors were highlighted, they were often followed by anecdotes and personal stories that discredited these behaviours as risk factors:

Emma: My aunty last year died of a brain tumour; she was a complete fitness freak, she hardly drank, she didn’t smoke, she’s never smoked, she ate real healthy, she was a proper fitness freak and she died of a brain tumour. (Focus Group 1 – Bristol)

Faith: Everything is related to cancer so in a way, if you’re that worried about it you’d stop doing a lot of things wouldn’t you? Because a lot of things are related to cancer

Hailey: You wouldn’t go out. (Focus Group 1 – Bristol)

There was a common misconception among many young mothers that cancer is a non-modifiable condition. This may be due to the rationalisation of cancer as a condition affecting ‘everyone’ – young and old, those leading healthy lifestyles and those leading unhealthy lifestyles, for example. In instances where some participants felt lifestyle behaviours might contribute to the development of cancer, there was an overarching feeling that if certain lifestyle behaviour choices do increase cancer risk, this should not limit their enjoyment of these behaviours; it was also suggested that since cancer is caused by many things other than behaviour, there is no justification in making changes. Correcting these misconceptions through appropriate education, which highlights the key risk factors for the development of cancer and considered approaches for behaviour modification, is imperative to future cancer prevention strategies.

#### Cancer prevention messages

The preventability of cancer through lifestyle choices was questioned by many of the participants and, as a result, there seemed to be mistrust around cancer prevention messages, particularly those which use shocking imagery or statistics as a tool to discourage behaviours such as smoking:

Amy: If it’s that bad, then why do the shops sell it?

Interviewer: So, do you think the advertising overstate the risk?

Amy: They do it so extreme that you just think, ‘No, it’s not …’ Like that’s not actually what’s going to happen … like they say every time you have a drag of a fag you mutate like. [Laughter] If you did that you’d be like proper mutated by now, wouldn’t you. Like, you’d be dead by now if you’d mutate that much. (Focus Group 1 – Bristol)

## Discussion

Findings from this study contribute to the literature on health behaviours, specifically cancer prevention, in young mothers. Using a multiple focus group, photo-elicitation aided, research methodology we were able to explore health behaviours of young mothers and the factors that influence their lifestyle choices, from their own perspectives, and link this to wider structure of inequality and social determinants of health.

Despite the increased risks of poor health behaviours, this ‘harder to reach’ group is often overlooked, with research either focusing on their young children or bypassing them altogether, further compounding health inequality and disadvantage. By engaging with a group of young mothers from across the United Kingdom, and allowing them to take photographs of things that mattered to them and shape the research, we were able to understand health behaviours and beliefs about cancer in a way that, to our knowledge, had not been done before.

Overall, participants demonstrated an accurate understanding of what behaviours define an individual as ‘healthy’; however, there was a general consensus that they themselves do not meet these criteria. There was a general lack of understanding as to how their behaviours influence cancer risk and any desire to modify lifestyle behaviours – such as eating healthier and increasing physical activity – focus on aesthetics and body image rather than health improvement or risk prevention.

Tackling the barriers to, and utilising the facilitators of, positive health behaviours is imperative to encouraging behaviour change in this population. Key barriers to positive health behaviours included (1) the cost of healthy foods and gym memberships, (2) the availability of convenience foods and takeaways, (3) lack of cooking skills, (4) lack of childcare and (5) the use of unhealthy behaviours such as smoking as a coping mechanism for stress.

Facilitators of positive health behaviours were (1) the use of social media, which is increasingly used for the delivery of health information and is delivered on a platform regularly accessed by members of this population (Chou et al., 2009); (2) commercial weight loss programmes, which encouraged weight loss and healthier lifestyles, via social support and the provision of healthy recipe ideas (Pallister et al., 2009); and (3) the social support from local community organisations, particularly those involved in this research.

Peer and social support, particularly from community centres and friendship networks, appears to be fundamental in the behavioural and lifestyle choices of this population. Future interventions should focus on strengthening variables such as self-efficacy, knowledge and social cohesion, which are known to enhance social support and encourage behaviour change (Cohen et al., 2000).

Cancer prevention knowledge was generally poor. Participants felt that cancer is not preventable and is associated with ‘bad luck’ rather than lifestyle choices. This was justified through lay epidemiological framings of personal experiences of ‘healthy’ family members getting cancer. This highlights a need for improved tailored education to appropriately explain personal cancer risk and to provide a better understanding of why ‘healthy’ people still get cancer. Further to this, there is a need to engage in debate about why ‘risky products’, most notably cigarettes, are legally available, given the dangers they present to health. Encouraging the use of reputable information sources, such as those provided by government and health bodies, is important to informing cancer prevention and health knowledge, particularly when using online resources as the quality and accuracy of information may be questionable (Eysenbach et al., 2002).

Evidence suggests that public health campaigns have been effective in informing public knowledge of the health risks associated with smoking (Redeker et al., 2009); yet, half of study participants continued to smoke. Tackling the use of smoking as a coping mechanism for stress in this population is vital to encourage cessation and reducing cancer risk in this group (Laurier et al., 2000). The delivery of health promotion messages is also important; participants felt that shock tactics were extreme and, as a result, disregarded the message as unbelievable; similarly in a UK sample of adolescents without children, pictorial warnings did not deter regular smokers (Moodie et al., 2015). Evidence suggests that the effectiveness of health messages delivered via mass media may be dependent on the socioeconomic status and the education of the target population (Niederdeppe et al., 2008), thus highlighting the need for tailored health promotion messages, particularly in harder-to-reach populations.

This study has highlighted the need for more focused campaigns and clearer links between health behaviours such as obesity, physical inactivity, alcohol consumption, poor dietary choices and cancer risk. Targeting these behaviours has the potential to have a multigenerational impact, as positive behaviours in parents will likely influence those of their children (Savage et al., 2007), and behaviours that begin in childhood are often carried on to adulthood (Whitaker et al., 1997). Addressing the consumption of takeaway and convenience foods consumed in this population is also imperative; interventions to promote cooking skills have proven beneficial at improving dietary habits and reducing the consumption of convenience foods in healthy adults of a low socioeconomic status (Reicks et al., 2014).

While interventions such as these could be valuable in encouraging healthier individual or collective behaviours, the wider context of inequality and multiple deprivations in which our participants lived need to be borne in mind. We have demonstrated in this study the ways in which broad structures of inequality mediate everyday health-related decisions and while targeting these everyday decisions may go some way to addressing the issue, we contend that it is these broad structures of deprivation, which need to be tackled.

The focus group and photo-elicitation aided approach which invited participants to capture ‘a week in your life’ was an informal and useful tool encouraging engagement between the group and with the researchers; this facilitated in-depth discussions and detailed accounts of lifestyle behaviour choices and factors which encourage or inhibit positive health behaviours. Participants led the conversations based on the photographs they provided; this encouraged detailed discussion of information relating to personal behaviours.

### Limitations

Due to time limitations of the project, participant numbers were smaller than anticipated at each individual site. However, we combined data across the three sites, analysing data from 27 participants in total, which provided sufficient rich, qualitative data to meet the research aims and reach data saturation.

As we recruited via health and community organisations, it is possible that we were unable to reach the ‘hardest to reach’ population as, by definition, its members may not be accessing these organisations. This is an ongoing issue in research of this type.

Finally, while the methods of focus groups and photo-elicitation enabled us to gather detailed information about participants’ everyday lives, there remains scope for exploring alternative methodological approaches. For example, future research may explore the use of body-mounted cameras to ‘follow’ participants everyday lives, which could capture movement through time and space and not rely on static images and retrospectively generated narratives.

## Conclusion

Study results provide a better understanding of the lifestyle behaviours of this population and identify some of the key challenges faced by young mothers in terms of following healthy lifestyle practices. The findings offer an evidence base to assist researchers working with this population who can use these in the development of practical interventions to encourage lifestyle change in this population. Such interventions have the potential to improve overall health and future health outcomes.

The data collected helped to illustrate members of this population’s health perceptions in relation to cancer risk; these also provided an understanding of how daily routines and circumstances influenced decisions and lifestyle behaviour choices. Beyond this, these also highlighted clear barriers to, and facilitators of, positive health behaviours. The methodology used was engaging and allowed for participant-led discussions. These insights should be utilised when developing future lifestyle, and cancer prevention, interventions in this population.

There are significant implications for health promotion and cancer prevention. First, there needs to be more debate and discussion around what counts as health and where/how people do/should get health messages. Second, interventions need to focus on the ‘everyday’ and, in line with this, keep intentions realistic. Further to this, interventions also need to look at the ways in which the broad structural inequalities that organise health and our relationships with each other are played out in this ‘everyday’ context. Finally, it needs to be acknowledged that the types of organisations involved in this study are excellent resources for young mothers and other marginalised groups and often support these individuals in accessing advice from health professionals such as GPs, instead of having them revert to asking their friends on Facebook. Future programmes and interventions should look to make use of these services rather than automatically implementing initiatives in places like GP surgeries and schools which may be viewed by these marginalised groups in a negative light; doing this, however, relies on stable and sustainable funding.

## Notes

1. Interviews in Belfast were primarily conducted by R.O.N., Bristol by L.H.-M. and in the Middlesbrough area by K.J.
